# Evaluating the E-Health Cloud Computing Systems Adoption in Taiwan’s Healthcare Industry

**DOI:** 10.3390/life11040310

**Published:** 2021-04-02

**Authors:** Shih-Chia Chang, Ming-Tsang Lu, Tzu-Hui Pan, Chiao-Shan Chen

**Affiliations:** 1 College of Management, National Taipei University of Business, Taipei 100025, Taiwan; chang@ntub.edu.tw; 2 Department of Business Administration, National Taipei University of Business, Taipei 100025, Taiwan; thpan880326@ntub.edu.tw; 3 Research Center for Healthcare Industry Innovation, National Taipei University of Nursing and Health Sciences, Taipei 112, Taiwan

**Keywords:** e-health (electronic health), cloud computing system, healthcare industry, multiple criteria decision-making (MCDM)

## Abstract

Although the electronic health (e-health) cloud computing system is a promising innovation, its adoption in the healthcare industry has been slow. This study investigated the adoption of e-health cloud computing systems in the healthcare industry and considered security functions, management, cloud service delivery, and cloud software for e-health cloud computing systems. Although numerous studies have determined factors affecting e-health cloud computing systems, few comprehensive reviews of factors and their relations have been conducted. Therefore, this study investigated the relations between the factors affecting e-health cloud computing systems by using a multiple criteria decision-making technique, in which decision-making trial and evaluation laboratory (DEMATEL), DANP (DEMATEL-based Analytic Network Process), and modified VIKOR (VlseKriterijumska Optimizacija I Kompromisno Resenje) approaches were combined. The intended level of adoption of an e-health cloud computing system could be determined by using the proposed approach. The results of a case study performed on the Taiwanese healthcare industry indicated that the cloud management function must be primarily enhanced and that cost effectiveness is the most significant factor in the adoption of e-health cloud computing. This result is valuable for allocating resources to decrease performance gaps in the Taiwanese healthcare industry.

## 1. Introduction

Electronic health (e-health) cloud computing is a progressive technology that can revolutionize the healthcare industry. Cloud computing has numerous advantages such as high speed, flexibility, scalability, fast deployment, resource sharing, energy saving, and cost-effective infrastructure, which can considerably influence daily life [1]. Computing facilities and resources can be established conveniently in any location. The healthcare industry is focused on sustainable development, and future healthcare models are expected to be data centric. Cloud computing can facilitate coordination, communication, and collaboration between various healthcare systems [2].

The rapid development of information and technology has resulted in advances in numerous healthcare applications such as hospital information systems, medical diagnostic systems, electronic health records, and healthcare monitoring [3,4]. Improving the efficiency and accuracy of healthcare information systems requires critical decision-making. Cloud computing is a novel technology that combines storage resource assignment and computing technology. The market of cloud computing is developing steadily and is prospective to be valued at approximately US$225 billion by 2020 [5]. Companies such as Aetna and IBM’s Active Health Management subsidiary have established novel clinical data management systems according to the structure of cloud computing [6]. The aim of the collaborative care solution is to provide controlled access to various types of clinical patient data from various sources, such as medication records, lab data, and claims as well as electronic medical records [1,7,8]. Microsoft and Google services such as Microsoft HealthVault and Google Health focus on novel services for storing medical records [7,8].

E-health cloud computing systems are preferable to e-health systems because of their agility and portability, and because they can be applied in remote areas with limited access to general medical services. E-health cloud computing systems are a low-cost method for transporting health services. Although numerous studies have been conducted on e-health cloud computing systems globally, the effectiveness of e-health cloud computing systems remains widely debated [8,9,10,11]. Nevertheless, e-health cloud computing system applications may revolutionize the healthcare industry in the future. Marcolino et al. [12] studied this efficiency of e-health cloud computing systems for managing illnesses and revealed mixed results. However, e-health cloud computing systems could be used to develop complementary devices for handling health problems in the future. E-health cloud computing systems can decrease healthcare service costs [13], and they can augment healthcare services by sending health-associated data to various departments [14,15]. Moreover, e-health cloud computing systems can be used to develop supportive devices that provide answers to health questions.

Studies on e-health cloud computing systems have focused on the following aspects: (1) evolving novel applications associated to fitness and health; (2) technology adoption of e-health cloud computing systems; and (3) clinical trials of e-health cloud computing system adoption [13,16,17,18]. However, few studies have focused on developing a context for evaluating this adoption of e-health cloud computing systems. Because of the widespread application of the cloud services and internet, the decision to adopt e-health cloud computing systems is critical.

Furthermore, cloud computing services are in the conceptualization phases at most medical organizations in Taiwan. Transitioning from conventional services to a cloud computing models is challenging for the healthcare industry. Therefore, the development of a systematic estimation model for medical institutions would promote the design systems of cloud computing and the prioritization and ranking of administration factors. Such an estimation model could also provide support to managers in administrative tasks, improve the effectiveness of cloud computing systems, indicate a suitable assignment of limited resources, and avoid needless resource waste and replacement.

Currently, although the healthcare industry is aggressively implementing cloud computing applications [19,20,21], a proper structure to evaluate the effectiveness of their adoption in the marketplace has received little attention. To satisfy these research gaps, this paper proposed a combined multicriteria decision making (MCDM) model to build an influential relations map (IRM) that combines DEMATEL (decision-making trial and evaluation laboratory) and DANP (DEMATEL-based Analytic Network Process) to investigate the influence weights of factors. The weights of the modified VIKOR (VlseKriterijumska Optimizacija I Kompromisno Resenje) technique were considered to integrate and estimate the factors of these gaps (financial performance & business performance) and the gaps were reduced based on IRM for evaluating cloud e-health system adoption. The purpose of this study is not to offer a multifaceted MCDM structure relating multiplex mathematical operations and functions, but, according to the empirical outcomes, these administrators can adopt approaches to successfully devote limited resources on suitable events for evaluating the model of cloud e-health system adoption.

The contributions of this paper are as follows: The adoption of e-health cloud computing systems is considered a decision-making concern with interaction and dependence factors. Interviews with experts and literature reviews were performed to generate 12 factors and four dimensions of e-health cloud computing functions to evaluate the e-health cloud computing system adoption for the healthcare industry. Next, we combined DEMATEL, DANP, and modified VIKOR to enhance an e-health cloud computing system adoption assessment model, which prioritizes the influence weights of e-health cloud computing function dimensions and factors and determines the obstacles during the adoption of e-health cloud computing systems. This proposed techniques not only address the dependence and interaction of factors but also provide valued data to establish an effect and cause map for strategy formation. Finally, the investigation of the assessment outcomes provides guidance to healthcare managers for detecting crucial factors influencing weights or gap assessments and provides the optimal method to enhance the existing adoption of e-health cloud computing systems.

This research is systematized as follows: Section 2 states these corresponding literatures relevant to the approaches and applications of e-health cloud computing system selection. Section 3 shows the planned approaches. It clarifies the numerical instance of the illustrated process in Section 4. Finally, it concludes the paper in Section 5. 

## 2. Literature Review

### 2.1. Applications in E-Health Cloud Computing Systems

Cloud computing which is applied for enhancing patient care is called e-health cloud computing, and it offers chances to address some limitations of hospital information systems [22,23]. The ability to access medical records anywhere and anytime can considerably improve healthcare services. Cloud computing can be used to access medical records and therefore improve healthcare facilitates. Because cloud computing is frequently operated in a shared and open environment, it is susceptible to malicious data thefts, attacks, and information losses. Security concerns are detrimental to the implementation of cloud computing systems in the healthcare industry. Healthcare specialists are distrustful of cloud computing because hackers may be able to access patient medical records. Thus, security and privacy concerns are valid [22,23]. Cloud computing service providers should address security concerns to improve trust among healthcare providers and patients [24,25]. A review verified that a growing number of healthcare administrations plan to implement cloud computing services to exploit cloud computing [26]. However, as mentioned previously, the adoption of cloud computing technology has presented numerous applications for the healthcare industry, principally with respect to their management (e.g., information integration, convenient information sharing, and cost effectiveness), service delivery (e.g., testing and debugging, software research and development, and system stability), security (e.g., confidentiality, data integrity, and access control), and software (e.g., software convenience, cloud-based medical image exchange, and software scalability) [18,24,25,27]. The healthcare industry is highly complex because of the operational difficulties of providing medical services [25,27,28]. Healthcare includes dissimilar stakeholders with various interests and industry-specific features [27,29]. Hence, cloud computing in healthcare is multifaceted, and its application via healthcare institutions can be ensured only under certain requirements [19]. When implementing cloud computing systems (i.e., making decisions about e-health cloud computing system adoption), numerous determinant problems associated with these conditions should be considered. An implementation decision without critical thought on the determining factors can not only hinder the productive application of cloud computing in the healthcare industry but also present problems. For example, an uninformed cloud computing adoption decision via a huge UK hospital did not provide the expected benefits and resulted in a financial deficit of £8.6 million and the temporary disruption of medical services [30,31].

E-health cloud computing systems are revolutionizing healthcare by providing users with novel protocols, which were not possible a decade ago. Innovative e-health cloud computing systems will finally become the novel cornerstone of modern health systems. Ambarkar and Shekokar [32] analyzed the security measures incorporated in the healthcare system. Farahani et al. [33] presented the benefits of implementing e-health cloud computing systems to enhance diagnostics in various applications. Cloud computing devices provide physicians with data-driven treatment plans to improve effective recovery. Abouelmehdi et al. [34] used big data to drive e-health cloud computing systems, personal health management, and knowledge discovery. They investigated the advantages and disadvantages of e-health cloud computing systems with regard to privacy and security technologies. Liao and Qiu [24] indicated that healthcare cloud management, cloud service delivery, and cloud software issues can influence the evaluation of cloud computing systems for healthcare industry. They proposed the practical application of a cloud computing-based approach for addressing healthcare issues. Hathaliya and Tanwar [23] offered a detailed literature review and investigation on the privacy and security problems in healthcare. Similarly, Semantha et al. [35] studied the current literature on privacy design in the healthcare industry and classified the limitations of the designs in the healthcare sector. They also provided crucial development directions for future study. 

After literature review, we discuss with administrators and experts in depth on the factors influencing the cloud computing system adoption in Taiwan’s healthcare industry. We summarized the factors which including cloud management, cloud service delivery, cloud security, and cloud software as our decision-making model. We discussed the detailed information in Section 2.2.

### 2.2. The Factors Influencing the Cloud Computing System Adoption

#### 2.2.1. Cloud Management

The adoption of cloud computing is partly driven by management concerns including information integration, information sharing, and cost effectiveness [26]. Implementing a system of cloud computing can cause considerable savings; the more expenditures a system saves with cloud computing, the greater is the effectiveness of the system—such as, when using software as a service—companies usually purchase version updates and pay monthly software fees based on the numeral of users, thereby decreasing information technology expenses and information integration. Therefore, if healthcare industry pays license fees by monthly, they could extend the of product lifecycle of software without bearing the risks of product redundancy or being bound via the models of traditional software licensing. Furthermore, service providers supply unlimited client services, pay-per-use, and dynamically adjustable via saving consumers the expenses, building virtual resource pools of maintaining, adopting, designing, and integrating infrastructures of customization. The services of the cloud also remove the expenditures related to buying data storage devices, providing management and maintenance, enabling convenient information sharing, and purchasing other hardware. Without cloud services, customers would need to solve these management problems [24,25]. Therefore, information integration, convenient information sharing, and cost effectiveness are serious concerns in the assessment of cloud management functions for e-health cloud computing systems.

#### 2.2.2. Cloud Service Delivery

Steady high-quality systems of cloud computing yield trustworthy services. Moreover, testing and debugging, software research and development, and system stability are closely related with system security owing to the degree to which system engineers stress that information security is related to the probability of realizing the security of data storage and system stability. As the service systems of healthcare cloud computing operate on the structures of network, they are weak to viruses, information theft, and hackers [24,25]. Therefore, testing and debugging, software research and development, and system stability are serious factors in the evaluation of cloud service delivery for e-health cloud computing systems.

#### 2.2.3. Cloud Security

We focused on three critical factors, namely confidentiality, data integrity, and access control. Delegating control of data to the cloud enhances the data compromise risk, owing to the data becoming reachable to numerous parties. The increase in the number of devices, applications, and parties increases the risk of data compromise. Integrity ensures the health data offered to any entity or captured via a system are consistent and accurate through the intended information and not modified in any method [36,37,38,39,40,41,42,43,44]. E-health cloud requires high reliability. E-health cloud data and services must be no error. This control policy is set such that only an authorized practitioner or a trusted third party can obtain the patient’s medical records. Numerous resolutions have been presented to deal with these access and control security worries. Attribute-based and role-based access controls are the most prevalent models for healthcare cloud computing.

#### 2.2.4. Cloud Software

The software function forms the crux of all services in e-health cloud computing systems. In the healthcare industry, convenient software, cloud-based medical image exchange, and software scalability are mostly critical. Owing to software convenience, software design and software scalability should satisfy the demands of the environment of healthcare, healthcare personnel and patients should be able to configure and regulate the functions of systems; when such demands are satisfied, the resulting cloud computing systems of healthcare can yield high user fulfillment [24,25]. Cloud-based medical image exchange is a critical requirement because this function may focus on emergency care. Hence, the information systems of health should include the structures that enable smooth and rapid transmission of medical information and classification of the medical data or images of critical patients, thus enabling staff to efficiently flag emergencies.

Based on literature review, expert opinions, and in-depth discussions with administrators of the healthcare industry and researchers, we summarized the cloud computing functions and security of e-health cloud computing systems as having the following four aspects: cloud management, cloud service delivery, cloud security, and cloud software. Table 1 displays a comprehensive report of these elements. Cloud management is affected by three factors, namely information integration, convenient information sharing, and cost effectiveness. Cloud service delivery is affected by three factors, namely testing and debugging, software research and development, and system stability. Cloud security is affected by three factors, namely confidentiality, data integrity, and access control. Cloud software is affected by three factors, namely software convenience, cloud computing medical image exchange, and software scalability. This study investigated the relationships between various elements and determined the significance of the factors to provide guidance on attaining the desired financial and business performance.

## 3. Methodology

**Figure 1:**
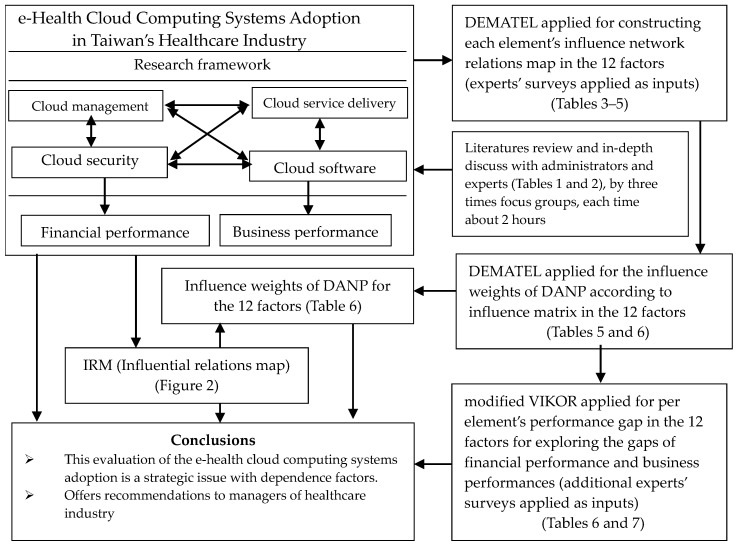
Modeling procedures of our proposed combined MCDM model.

In this research, we concisely illustrate the presented integrated MCDM model. Due to prior practice, this model is determined to be a suitable technique for estimating a strategy to ensure success when adopting an e-health cloud computing system. The present study integrated cloud management, cloud service delivery, cloud security, and cloud software. The combined model was used to address decision-making and assessment for e-health cloud computing system adoption problems. All infrastructural and assessment factors of e-health cloud computing systems and their relations with financial and business performance were investigated. The object of this research is to combine the model to provide ranking and selection, as well as enhanced performance. Finally, the main enhancement of our integrated model is its managerial ability in selection and enhancement; thus, this model can support healthcare managers to enhance their decision-making. This integrated model is separated into three parts [45,46,47,48,49,50]: (1) DEMATEL is used to build a structure with an influence relationship map (IRM) based on the factors/dimensions (the first survey: DEMATEL questionnaire). (2) DEMATEL-based ANP (DANP) is used to attain these influence weights (from the DEMATEL method and outcomes). (3) This modified VIKOR approach is applied to empirically estimate gap performance (financial and business performance) in the healthcare industry, which occur during e-health cloud computing system adoption (the second survey: performance questionnaire). Hence, this integrated method can help managers to define how to evaluate the adoption of an e-health cloud computing system, in order to achieve a good fit outcome, which is followed by IRM for each alternative. The development of the combined model is shown in Figure 1.

### 3.1. Data Collection

Through this literature review and 15 experts’ opinions with conducting focus groups at least three times, each time we spent about 2 h (we discussed the topic, the factors of influence on the e-health cloud computing systems adoption via literature review and discussed with administrators and experts in depth regarding the factors of influence in Taiwan’s healthcare industry in the real world). Based on the consensus via focus groups, we present four dimensions and confirm 12 factors of influence on the e-health cloud computing systems adoption. After that, we built up two sets of surveys of DEMATEL and performance questionnaires (see Appendix A and Appendix C stages). For the DEMATEL questionnaire, we used the five Likert scales, which are settled the scale through “very high influence (4)” to “lack of influence (0)”. In addition, for the performance questionnaire, we used the 11 Likert scales in which the scored responses range from 0 to 10: totally dissatisfied (0) to extremely satisfied (10). Finally, the two set of surveys of DEMATEL and performance questionnaires which are confirmed by the 15 experts. The study used the proposed approach in the healthcare industry in Taiwan as a case research. All data were collected from the 15 experts. To ensure the reliability of these experts, this research established personal interviews face-to-face to make sure that the definitions of the questions inquired could be realized. These experts are researchers and those at the upper administration level, with an average working experience in the healthcare industry of more than 10 years, and the related information of the specialists is shown as Table 2. They agreed that all elements are crucial and comprehensive, showing that the elements need to be implemented into this research. For the consistency (in consensus, see Table 3 in Note 1), the statistical significance confidence is 95.381%, in consensus, that is greater than 95%, and this error gap less than 5% is 4.619% in this study. As this consistency ratio is less than the significance value, the threshold is usually set to 5%—it can be determined that the 15 specialists’ replies have achieved a suitable consensus and consistency.

### 3.2. DEMATEL for Assembling an Evalution Structure through IRM

The DEMATEL technique was established via the Battelle Memorial Institute of Geneva from 1972 for the Science and Human Affairs Program. This objective was to investigate difficult and multifaceted real-life issues—for example, energy, ethnicity related problems, and environmental protections—and detect practical resolutions via a hierarchical construction [51,52]. This technique can effectually integrate professional domain knowhow and make clear the causal relations among influence elements. Moreover, regarding the causal relations between factors into an easy to understand systemic pattern, this technique uses a mathematical theory to calculate the degrees of indirect and direct influences per factor/dimension [45,46,47,48,49,50]. The most characteristic factor of the DEMATEL technique is its ability to demonstrate the relations among groups and acquire the key aspects of the demonstrative factors. In addition, DEMATEL has been effectively applied in numerous applications, for example, RFID adoption, legal AI Bot implementation, strategic orientations for promoting hotel services, and estimating sustainable development performance [48,53,54,55].

The DEMATEL method comprises four main stages: (1) using the direct relationship matrix for constructing this research framework; (2) acquiring a normalized matrix of direct relationship; (3) obtaining a matrix of full influence relationship; and (4) creating an influence relationship map (see Appendix A).

### 3.3. DANP Technique for Derviving Influence Weights 

DANP is used to determine this relationship matrix of full influence to acquire these interdependent relationship weights among the factors/dimensions via applying the procedures and conceptions of ANP [56]. The traditional approach that is using DEMATEL and ANP needs to apply both surveys. This survey of DEMATEL is primarily applied to decide the mutual influences between estimation attributes and set up a hierarchic structure; then, the ANP survey is used to acquire the weights of the factors and estimation outcomes. However, the DANP technique only needs the survey of DEMATEL [57]. This DANP’s weight value indicates the ratio of the factors/dimensions, as well as their influence degree on the full model, which is simultaneously according to the degrees of received and given effects in a circumstance. The DANP technique comprises three main stages: (1) emerging an unweighted super-matrix; (2) creating a super-matrix weight; and (3) assembling the supermatrix weight (see Appendix B).

### 3.4. Modified VIKOR Technique

The modified VIKOR technique was developed via Opricovic and Tzeng [58,59] as a technique of counting the factor score as the approach for the order of preference via similarity to an ideal solution (TOPSIS) was not available. These TOPSIS and VIKOR techniques are ranking approaches applied in MCDM. Both approaches utilize the conception of compromise to deal with the disadvantages and advantages of the estimation elements, and both rank the alternatives according to the nearness of a limited number of estimated elements to the ideal resolution. 

The TOPSIS technique ranks all resolutions via the distance through the negative ideal resolution and closeness to the positive ideal resolution. Nevertheless, as estimating two factors in cases when the scenarios fall diagonally on the plane, the TOPSIS technique cannot really reflect per scenario’s closeness to the ideal resolution, and the most optimal resolution cannot be determined. To avoid the defect in the TOPSIS technique, the modified VIKOR technique applies the aspired value and worst level to compare the alternatives estimation scores and detect an ideal resolution. Based on the positive ideal and negative ideal, as determined by a preference for remaining near the positive ideal point, that is the fundamental idea of conventional thought. This conception of gap estimates the nearness positive ideal point [45,46,47,48,49,50]. A modified VIKOR has been effectively applied in numerous applications, for example, B2B commerce implementation, RFID adoption, and Legal AI Bot implementation [53,54].

Modified VIKOR comprises these next stages: (1) invent the worst and the best values in evaluation factors. It shifts from selection and ranking as defining the best methods to performance enhancement of present approaches according to IRM; (2) analyze the mean of group utility and maximal regret; and (3) acquire the comprehensive indicator and sort out the outcomes (see Appendix C).

## 4. Case Analysis

In this study, we performed a case on the healthcare industry in Taiwan. This MCDM model was used to investigate the interdependence and feedback between the factors affecting performance when adopting an e-health cloud computing system for the healthcare industry in Taiwan. The results can help managers and decision-makers and strongly affects e-health cloud computing system adoption, particularly when healthcare resources are limited. The outcomes of the empirical case are presented along with related discussions.

### 4.1. Analytical Results

The DEMATEL assessment structure was developed for the e-health cloud computing systems with four dimensions and 12 factors. The factors and dimensions for the total influence matrix, as stated in Table 3 and Table 4, were attained through expert surveys. Experts’ views on the four dimensions were collected, and the relations between these spaces of impact associated with the other dimensions were determined (Table 4). 

Based on the summation of row and column aggregations means that any of the factor *x* effects on all other factors, named $b_{x}$, and *x* is influenced by all other factors, named $t_{x}$. For the total influence prominence $\left(b_{x}+t_{x}\right)$, the cloud management (*A*_1_) exhibited the most critical effect on the strength of the relationships. By contrast, cloud software (*A*_4_) exhibited the weakest effect. Based on this net influence $\left(b_{x}-t_{x}\right)$, cloud service delivery (*A*_2_) exhibited the most direct effects among the dimensions, and cloud security (*A*_3_) was the most vulnerable.

Table 4 displays the influence relationship factors, and Table 5 illustrates the relationships between the degrees of direct and indirect influences as well as comparisons with other factors. Information integration (*S*_1_) was the most critical factor for intelligent administration; software scalability (*S*_12_) had the weakest effect on other factors. According to Table 5, software research and development (*S*_5_) had the strongest influence on other factors, and cost effectiveness (*S*_3_) was the most influenced by other factors.

**Figure 2:**
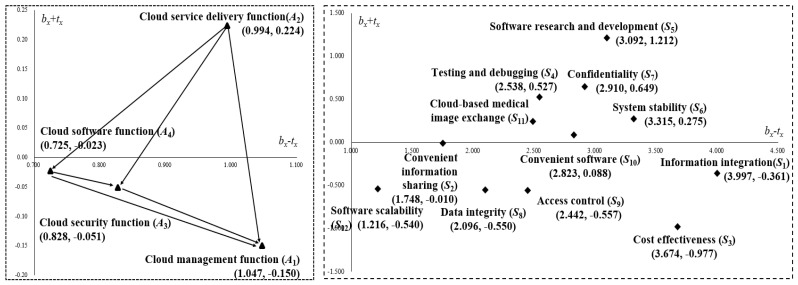
The influential relationship map (IRM) for dimension and factor separately.

By using the proposed DEMATEL model, this study determined the relationship between numerous factors and dimensions through IRM. Figure 2 illustrates that the cloud service delivery function (*A*_2_) influenced other aspects, namely, the cloud software (*A*_4_), cloud security (*A*_3_), and cloud management (*A*_1_), whereas cloud service delivery (*A*_2_) had the strongest effect on other dimensions.

Figure 2 displays the IRM of the 12 factors in four dimensions. Based on the cause-and-effect relation, Quadrant I in the map covers the main factors (*S*_5_, *S*_7_, *S*_4_, *S*_6_, *S*_11_, and *S*_10_) to be determined. These factors are critical for various approaches for the adoption of an e-health cloud computing system in the healthcare industry, and cover the driving factors (*S*_2_, *S*_1_, *S*_12_, *S*_8_, *S*_9_, and *S*_3_), which only affect other factors in Quadrant II. Therefore, regarding the relationship of factors, the most influenced factors are *S*_5_, *S*_7_, *S*_4_, *S*_6_, *S*_11_, *S*_10_, *S*_2_, *S*_1_, *S*_12_, *S*_8_, *S*_9_, and *S*_3_. Regarding software research and development (*S*_5_), the adoption of an e-health cloud computing system indicates operational applications in the healthcare industry. 

DANP was used to obtain the influence weights of the individual factors, as illustrated in Table 5 and Table 6. The outcomes demonstrated cost effectiveness (*S*_3_), information integration (*S*_1_), and access control (*S*_9_) are the critical factors for improving adoption. Furthermore, the weights of influence are combined with the DEMATEL technique to explain the sequence of decreasing these gaps, as determined through the IRM and modified VIKOR.

This study assessed e-health cloud computing system adoption, and investigated the effect of its adoption on financial (***&***_1_) and business performance (***&***_2_). Furthermore, the modified VIKOR approach was used to estimate and improve reliance. Table 6 illustrates the gap estimation for adopting an e-health cloud computing system through the modified VIKOR approach. Managers can distinguish questions based on the composite index according to holistic or individual dimensions for using these outcomes (see the Table 6). 

By applying the guidelines to these dimensions and factors, these gaps of performances can be prioritized and determined to achieve the desired level. Regarding financial performance (***&***_1_), cost effectiveness (*S*_3_) exhibited a maximum gap of 0.553 and was the initial factor to be enhanced, followed by system stability (*S*_6_) and software scalability (*S*_12_). Among all factors for financial performance (***&***_1_), administrators should focus on cost effectiveness (*S*_3_). Regarding business performance (***&***_2_), cost effectiveness (*S*_3_) exhibited the highest gap of 0.513 and was the main factor to be enhanced, followed by information integration (*S*_1_) and system stability (*S*_6_). For business performance (***&***_2_), administrators should focus on cost effectiveness (*S*_3_). These results indicated that a sequence array, from the most to the least significant, is required for all the factors to attain the desired level.

Improvement priorities can also be used in separate dimensions—for example, regarding financial performance (***&***_1_). Within cloud management (*A*_1_), the gap values are prioritized as follows: cost effectiveness (*S*_3_), information integration (*S*_1_), and convenient information sharing (*S*_2_). Within cloud service delivery (*A*_2_), the gap values are prioritized as follows: system stability (*S*_6_), testing and debugging (*S*_4_), and software research and development (*S*_5_). Regarding cloud security (*A*_3_), enhancement priorities are ordered as follows: confidentiality (*S*_7_), access control (*S*_9_), and data integrity (*S*_8_). Regarding cloud software (*A*_4_), enhancement priorities are ordered as follows: software scalability (*S*_12_), cloud-based medical image exchange (*S*_11_), and software convenience (*S*_10_). Regarding business performance (***&***_2_), enhancement priorities are ordered as follows: (*S*_3_), (*S*_1_), and (*S*_2_) within for cloud management (*A*_1_); (*S*_6_), (*S*_5_), and (*S*_4_) for cloud service delivery (*A*_2_); (*S*_9_), (*S*_7_), and (*S*_8_) for cloud security (*A*_3_); and (*S*_12_), (*S*_11_), and (*S*_10_) for cloud software (*A*_4_).

### 4.2. Discussion

Most approaches cannot determine the multifaceted interrelationships between the numerous dimensions and factors that influence the adoption of an e-health cloud computing system. This study distinguished the relationships between various dimensions and factors. Based on the matrix of total influence, cloud service delivery (*A*_2_) exhibited the greatest influence. According to this net effect, cloud service delivery (*A*_2_) also exhibited the strongest net effect on the other aspects. Managers in the healthcare industry should enhance cloud service delivery (*A*_2_) first, followed by cloud software (*A*_4_), cloud security (*A*_3_), and cloud management (*A*_1_) when enhancing system adoption.

The proposed framework involves cloud management (*A*_1_), cloud service delivery (*A*_2_), cloud security (*A*_3_), and cloud software (*A*_4_) based on the recommendations of experts regarding the factors affecting system adoption performance. According to recommendations by specialists in the healthcare industry, the recommended structure relates cloud management functions, cloud service delivery function, cloud security function, and cloud software function (A_4_) dimensions to investigate these factors impacting e-health cloud computing system adoption performance (see Figure 2).

These empirical outcomes also indicated the premeditated factors within the separate dimensions. Table 7 summarizes the sequence of influence factors in the separate dimensions according to the outcomes of DEMATEL and modified VIKOR. For cloud service delivery function (*A*_2_), software research and development (*S*_5_) were the most affected factors and required initial enhancement, followed by testing and debugging (*S*_4_), and system stability (*S*_6_). After using the gap values, as presented via the panel of specialists, crucial enhancement designs were comprehensively and uniquely considered in terms of both holistic and separate dimensions. For managers in the healthcare industry in Taiwan, understanding the improvement priorities for e-health cloud computing system adoption is critical. The results displayed in Table 7 indicate enhancement sequences that can be implemented to achieve the intended financial and business performance. For example, to decrease the performance gaps among the existing and aspired levels of financial performance, the sequence for enhancement is cloud management (*A*_1_), cloud software (*A*_4_), cloud service delivery (*A*_2_), and cloud security (*A*_3_). However, managers in the healthcare industry should be cautious because, when using the MCDM model, the significance of the 12 factors might change according to circumstances. Thus, administrators must identify gaps before making decisions.

## 5. Conclusions

This study proposed a MCDM technique in which DEMATEL, DANP, and the modified VIKOR approach were combined to estimate the interdependence and feedback between numerous features when adopting an e-health cloud computing system in the healthcare industry. Because the various healthcare departments have limited resources, in the suggested structure, cloud management, cloud service delivery, cloud security, and cloud software should be incorporated. According to these outcomes, cloud service delivery exhibited the highest net influence of 0.224, and thus should be enhanced initially. Cost effectiveness, which exhibited the maximum global weight of 0.144, is the most critical feature for improving system adoption performance in the healthcare industry in Taiwan. When scheduling the adoption of a cloud computing system, companies typically estimate their return on investment (ROI) to determine whether an adoption plan is suitable. Companies generally avoid adopting a system if the cost does not justify the expenses. Although healthcare institutions are not focused on profit, they must demonstrate that their decision-making procedures are similar to those of for-profit organizations regarding the assessment and planning of a service system for e-health cloud computing. Decision-makers need to carefully determine the cost savings that cloud computing services can yield. The costs of implementing an e-health cloud computing service system can be avoided if the ROI is not high. The gap values of total performance represent the ranges for enhancement, which are 0.382 for financial performance (***&***_1_) and 0.361 for business performance (***&***_2_). Cloud management (*A*_1_) exhibited the largest gap (0.420) in financial performance (***&***_1_) and the largest gap (0.425) in business performance (***&***_2_). Thus, cloud management should be enhanced first if managers hope to achieve the anticipated performance. Moreover, the results suggested that managers can select a suitable sequence of factors to enhance system adoption. 

The proposed method has several limitations. Firstly, the sample size in this study was limited; thus, a higher number of samples should be considered to obtain complementary explanatory abilities for multifaceted and specialized assessment research. Next, the outcomes obtained in this study should be verified with more samples. MCDM helps managers to understand the elements required to improve performance assessment. Finally, this is a case study in Taiwan that may not be applicable in other countries with different health systems and legislation. Future studies can consider additional multiple criteria approaches (for example, the best–worst method and outranking methods) to evaluate the influence of the weights of e-health cloud computing system adoption estimation. Finally, numerous approaches, such as longitudinal studies, can be used to determine the factors related to the adoption of an e-health cloud computing system in future research.

## Figures and Tables

**Table 1:** Explanation of dimensions and factors.

Dimensions/Factors		Descriptions
*A*_1_	Cloud management	
*S*_1_	Information integration	Cloud computing system adoption based on discussions with healthcare staff and retaining their individual knowledge to effectively discuss system demands.
*S*_2_	Convenient information sharing	To conveniently exchange records regarding relevant patient test reports and physiological conditions.
*S*_3_	Cost effectiveness	Savings achieved by designing and adopted cloud computing systems, such as reductions in software costs, hardware costs, and data storage costs.
*A*_2_	Cloud service delivery	
*S*_4_	Testing and debugging	Modification and testing of novel anti-intrusion programs, development of software, and establishment of routine operational controls.
*S*_5_	Software research and development	Software development and research for the cloud computing operations of the system.
*S*_6_	System stability	Comprehensive protection system and data security to prevent system failure, cyberattacks, and virus attacks.
*A*_3_	Cloud security	
*S*_7_	Confidentiality	Ensure that patient information remains undisclosed during transmissions between cloud computing and devices in the e-health system.
*S*_8_	Data integrity	Evaluation of the integrity of the nodes involved in the exchange between the receiver and sender.
*S*_9_	Access control	Restricting and controlling user access to protected personal information.
*A*_4_	Cloud software	
*S*_10_	Convenient software	Ensure that the functions of cloud computing software can be rapidly executed and configured and that no complex procedures or inputs are required.
*S*_11_	Cloud-based medical image exchange	Evolving cloud exchanges of medical images through the cloud-based e-health system.
*S*_12_	Software scalability	Ensure that the scalability of the software design satisfies user demands pertaining to healthcare.

**Table 2:** The information of experts.

Category	Number of Experts
Education Level	
Bachelor	1
Master	9
Ph.D.	5
Years of working experiences	
Between 10 and 14 years	1
Between 15 and 19 years	4
More than 20 years	10
Job Level	
Researchers in related industry	4
Top managers in healthcare	11

**Table 3:** Initial influence matrix for each factor.

Factors	*S*_1_	*S*_2_	*S*_3_	*S*_4_	*S*_5_	*S*_6_	*S*_7_	*S*_8_	*S*_9_	*S*_10_	*S*_11_	*S*_12_
*S*_1_	0.000	0.733	2.867	0.133	0.667	1.200	0.800	2.067	1.867	1.133	1.667	0.667
*S*_2_	1.533	0.000	2.600	0.133	0.133	0.133	0.467	0.200	0.200	0.200	0.200	0.200
*S*_3_	3.533	1.000	0.000	0.333	0.333	0.933	0.733	0.800	0.867	0.267	0.267	0.200
*S*_4_	0.467	1.067	1.200	0.000	2.067	2.200	0.733	0.867	0.867	0.400	0.400	0.133
*S*_5_	1.067	2.200	2.333	2.400	0.000	2.000	1.000	1.000	1.667	0.933	0.933	0.133
*S*_6_	1.467	0.400	2.533	1.067	1.067	0.000	0.133	0.267	0.267	3.200	0.667	2.200
*S*_7_	2.467	0.133	2.067	1.133	0.867	0.800	0.000	1.867	3.267	0.133	0.133	0.133
*S*_8_	0.667	0.133	1.467	0.667	0.133	0.133	0.667	0.000	1.267	0.133	0.133	0.133
*S*_9_	1.267	0.133	0.667	0.133	0.133	0.133	2.467	1.067	0.000	0.133	0.133	0.133
*S*_10_	1.667	0.200	0.400	0.400	0.667	2.200	0.133	0.133	0.133	0.000	3.000	1.200
*S*_11_	0.800	0.133	0.800	1.200	0.667	0.667	0.667	0.667	0.133	3.000	0.000	0.933
*S*_12_	0.133	0.133	0.133	0.133	0.133	0.800	0.133	0.133	0.133	0.267	0.133	0.000

Note 1: It’s a k×k matrix and n=12. And $\frac{1}{k^{2}}\sum_{i=1}^{k}\sum_{j=1}^{k}\frac{\left|t_{ij}^{n}-t_{ij}^{n-1}\right|}{t_{ij}^{n}}\times 100\%=$ 4.619% < 5%, and significant confidence is 95.381%, as n=15 represents the number of experts and $t_{ij}^{n}$ is the average impact of i factor on j; and *n* represents number of factors.

**Table 4:** Summation of effects on dimensions and total influence matrix.

Dimensions	*A*_1_	*A*_2_	*A*_3_	*A*_4_	$b_{x}$	$t_{x}$	$b_{x}+t_{x}$	$b_{x}-t_{x}$
Cloud management function (*A*_1_)	0.167	0.077	0.119	0.085	0.448	0.598	1.047	−0.150
Cloud service delivery function (*A*_2_)	0.202	0.146	0.127	0.134	0.609	0.385	0.994	0.224
Cloud security function (*A*_3_)	0.135	0.071	0.134	0.048	0.388	0.439	0.828	−0.051
Cloud software function (*A*_4_)	0.094	0.091	0.059	0.107	0.351	0.374	0.725	−0.023

**Table 5:** Sum of effects of each factor, weight and ranking.

Dimensions/Factors	$b_{x}$	$t_{x}$	$b_{x}+t_{x}$	$b_{x}-t_{x}$	Influential Weights (Global Weights)
Cloud management function (*A*_1_)					0.337
Information integration (*S*_1_)	1.818	2.179	3.997	−0.361	0.140
Convenient information sharing (*S*_2_)	0.869	0.879	1.748	−0.010	0.053
Cost effectiveness (*S*_3_)	1.349	2.326	3.674	−0.977	0.144
Cloud service delivery function (*A*_2_)					0.207
Testing and debugging (*S*_4_)	1.533	1.006	2.538	0.527	0.059
Software research and development (*S*_5_)	2.152	0.940	3.092	1.212	0.056
System stability (*S*_6_)	1.795	1.520	3.315	0.275	0.091
Cloud security function (*A*_3_)					0.254
Confidentiality (*S*_7_)	1.779	1.131	2.910	0.649	0.076
Data integrity (*S*_8_)	0.773	1.323	2.096	−0.550	0.083
Access control (*S*_9_)	0.942	1.499	2.442	−0.557	0.095
Cloud software function (*A*_4_)					0.202
Convenient software (*S*_10_)	1.455	1.367	2.823	0.088	0.082
Cloud-based medical image exchange (*S*_11_)	1.365	1.121	2.486	0.244	0.069
Software scalability (*S*_12_)	0.338	0.878	1.216	−0.540	0.052

**Table 6:** Gap assessment of e-health cloud computing systems adoption via modified VIKOR.

Dimensions/Factors	Local Weight	Global Weight	Gap of Performance	
			(*&*_1_)	(*&*_2_)
Cloud management function (*A*_1_)		0.253	0.420	0.425
Information integration (*S*_1_)	0.417	0.140(2)	0.333	0.413(2)
Convenient information sharing (*S*_2_)	0.157	0.053	0.287	0.213
Cost effectiveness (*S*_3_)	0.426	0.144(1)	0.553(1)	0.513(1)
Cloud service delivery function (*A*_2_)		0.265	0.366	0.352
Testing and debugging (*S*_4_)	0.286	0.059	0.300	0.233
Software research and development (*S*_5_)	0.271	0.056	0.293	0.387
System stability (*S*_6_)	0.443	0.091	0.453(2)	0.407(3)
Cloud security function (*A*_3_)		0.232	0.345	0.374
Confidentiality (*S*_7_)	0.299	0.076	0.400	0.373
Data integrity (*S*_8_)	0.328	0.083	0.287	0.367
Access control (*S*_9_)	0.373	0.095(3)	0.353	0.380
Cloud software function (*A*_4_)		0.250	0.379	0.250
Convenient software (*S*_10_)	0.403	0.082	0.353	0.167
Cloud-based medical image exchange (*S*_11_)	0.341	0.069	0.380	0.300
Software scalability (*S*_12_)	0.256	0.052	0.420(3)	0.313
	Total gap (*S_Q_*)		0.382	0.361

**Table 7:** Sequence of enhancement priority for the strategy.

Formula	Sequence of Enhancement Priority	
F1: Influence network of dimensions	(*A*_2_), (*A*_4_), (*A*_3_), (*A*_1_)	
F2: Influence network of factors within individual dimensions	(*A*_1_): (*S*_2_), (*S*_1_), (*S*_3_)	
	(*A*_2_): (*S*_5_), (*S*_4_), (*S*_6_)	
	(*A*_3_): (*S*_7_), (*S*_8_), (*S*_9_)	
	(*A*_4_): (*S*_11_), (*S*_10_), (*S*_12_)	
F3: Sequence of dimension to rise to aspired value in the two e-health cloud computing systems adoption performance (by gap value)	Financial performance (*&*_1_)	(*A*_1_), (*A*_4_), (*A*_2_), (*A*_3_)
	Business performance (*&*_2_)	(*A*_1_), (*A*_3_), (*A*_2_), (*A*_4_)
F4: Sequence of factors to rise to aspired value within individual dimension in the two e-health cloud computing systems adoption performance (from high to low)	Financial performance (*&*_1_)	
	(*A*_1_): (*S*_3_), (*I*_1_), (*S*_2_) (*A*_2_): (*S*_6_), (*I*_4_), (*I*_5_) (*A*_3_): (*S*_7_), (*S*_9_), (*S*_8_) (*A*_4_): (*I*_12_), (*I*_11_), (*I*_10_)	
	Business performance (*&*_2_)	
	(*A*_1_): (*S*_3_), (*S*_1_), (*S*_2_) (*A*_2_): (*S*_6_), (*S*_5_), (*S*_4_) (*A*_3_): (*S*_9_), (*S*_7_), (*S*_8_) (*A*_4_): (*S*_12_), (*S*_11_), (*S*_10_)	

## Data Availability

Not applicable.

## Appendix A. DEMATEL Technique

The technique of DEMATEL has four stages, as follows:

### Appendix A.1. Stage 1: Using the Direct-Relation Matrix for Constructing the Research Framework

As the (z) factors in an estimation structure have been established, this regular influence degree scale is settled (scale through “very high influence (4)” to “lack of influence (0)”). These measured degrees of effect among the dimensions or factors are applied via natural language. The regular of n specialists apply a regular scale to demonstrate the direct influence degree through factor/dimension *x* on factor/dimension *y* in matrix $V={\left[v_{xy}\right]}_{z\times z}={\left[(\sum_{a=1}^{n}p_{xy}^{a})/n\right]}_{z\times z}$ (in which $p_{xy}\neq 0$; otherwise, $p_{xy}=0$, and n is the amount of specialists). Lastly, the mean is applied to assimilate a major matrix $Z={\left[z_{xy}\right]}_{a\times a}$ of the direct relationship, which demonstrate the actual practices of the experts.

### Appendix A.2. Stage 2: Acquiring a Normalized Matrix of the Direct Relationship

A normalized major direct relationship matrix *P* is reached through normalizing this major direct relationship matrix *U* uses Equations (A1) and (A2) where all the matrix *O* diagonal terms are 0, and the maximum summation of each column and row is 1:

(A1) $$\eta =\underset{x,y}{\max}\left[\max_{x}\sum_{y=1}^{u}\left|z_{xy}\right|,\max_{y}\sum_{x=1}^{u}\left|z_{xy}\right|\right]$$

(A2) P=Z/η

### Appendix A.3. Stage 3: Obtaining a Matrix of Full Influence Relationship

This matrix ***O*** has a full influence relationship, which is obtained by Equation (A3). It is able to provide assurances of convergent determinations to the matrix reversal in a method similar to capturing the matrix of a Markov chain. Therefore, the full influence relationship matrix ***O*** can be obtained from the values in the normalized matrix ***P*** of the direct relationship, and ***L*** is the individuality matrix, as follows:

(A3) $$O=P+P^{2}+\cdots +P^{h}=P{\left(L-P\right)}^{-1},\;\mathrm{as}\;\lim_{h\to \infty }P^{h}={\left[0\right]}_{z\times z}$$

The matrix ***O*** of the full influence relationship can be obtained by ***O_k_*** and ***O_V_*** is based on a hierarchical framework, as shown by Equations (A4) and (A5), respectively.

(A4) $$O_{k}=\begin{array}{ccc} & & \begin{array}{ccccc} V_{1} & & V_{y} & & V_{m}\\ k_{11}\ldots k_{1m_{1}} & \cdots & k_{y1}\ldots k_{ym_{y}} & \cdots & k_{m1}\ldots k_{mm_{m}} \end{array}\\ \begin{array}{c} V_{1}\\ \vdots \\ V_{x}\\ \vdots \\ V_{m} \end{array} & \begin{array}{c} k_{11}\\ k_{12}\\ \vdots \\ k_{1m1}\\ \vdots \\ k_{x1}\\ k_{x2}\\ \vdots \\ k_{xm_{x}}\\ \vdots \\ k_{m1}\\ k_{m2}\\ \vdots \\ k_{mm_{m}} \end{array} & {\left[\begin{array}{ccccc} O_{k}^{11} & \cdots & O_{k}^{1y} & \cdots & O_{k}^{1m}\\ \vdots & & \vdots & & \vdots \\ O_{k}^{x1} & \cdots & O_{k}^{xy} & \cdots & O_{k}^{im}\\ \vdots & & \vdots & & \vdots \\ O_{k}^{m1} & \cdots & O_{k}^{my} & \cdots & O_{k}^{mm} \end{array}\right]}_{z\times z|m<z,\sum_{y=1}^{m}m_{y}=z} \end{array}$$

(A5) $$O_{V}={\left[\begin{array}{ccccc} o_{11} & \cdots & o_{1y} & \cdots & o_{1m}\\ \vdots & & \vdots & & \vdots \\ o_{x1} & \cdots & o_{xy} & \cdots & o_{xm}\\ \vdots & & \vdots & & \vdots \\ o_{m1} & \cdots & o_{my} & \cdots & o_{mm} \end{array}\right]}_{m\times m}$$

### Appendix A.4. Stage 4: Creating an Influence Relationship Map

By summing the separate rows and columns of this full influence relationship matrix ***P***, we obtain the summation of the vectors by all rows and columns, as shown below:

(A6) $$b_{x}={\left[\sum_{y=1}^{z}p_{xy}\right]}_{z\times 1}^{\prime },x\in \{1,2,\ldots ,z\}$$

(A7) $$t_{y}={\left[\sum_{x=1}^{z}o_{xy}\right]}_{1\times z},y\in \{1,2,\ldots ,z\}$$

As y=x (i.e., the summation of row and column aggregations means that any of the factor *x* effects on all other factors, named $b_{x}$, and *x* is influenced by all other factors, named $t_{x}$), this value $\left(b_{x}+t_{x}\right)$ illustrates the total influences given and received via enabler factor x (i.e., representing the degree of effect that the enabler factor x plays in the entire system, also called “prominence”). Moreover, the value $\left(b_{x}-t_{x}\right)$ indicates the clear influence of enabler x on this entire method. If $\left(b_{x}-t_{x}\right)$ is a positive value, then x belongs to the net cause group. Otherwise, if $\left(b_{x}-t_{x}\right)$ is a negative value, then x belongs to the net effect group. Hence, via drawing the dataset of the ($b_{x}+t_{x}$,$b_{x}-t_{x}$), we obtain the IRM of the dimensions and factors.

## Appendix B. DANP Technique

This DANP technique contains three key stages, as follows:

### Appendix B.1. Stage 1: Emerging an Unweighted Super-Matrix

The emerging supermatrix $W={\left(O_{k}^{b}\right)}^{\prime }$ is unweighted, and can be separated into two stages: the initial act normalizes matrix $O_{k}$ with the full influence relationship to attain the normalized full-influence relationship matrix $O_{k}^{b}$, while the second act transposes the normalized matrix $O_{k}^{b}$ full influence relationship to attain $W={\left(O_{k}^{b}\right)}^{\prime }$.

This normalized matrix $O_{k}^{b}$ of the full influence relationship (Equation (A9)) is attained via separately normalizing the rows of the dimensions in the full relationship matrix $O_{k}$ (Equation (A8)), and the summation of the separate rows equals the number of dimensions:

(A8) $$O_{k}^{b}=\begin{array}{ccc} & & \begin{array}{ccccc} V_{1} & & V_{y} & & V_{m}\\ k_{11}\ldots k_{1m_{1}} & \cdots & k_{y1}\ldots k_{ym_{y}} & \cdots & k_{m1}\ldots k_{mm_{m}} \end{array}\\ \begin{array}{c} V_{1}\\ \vdots \\ V_{x}\\ \vdots \\ V_{m} \end{array} & \begin{array}{c} k_{11}\\ k_{12}\\ \vdots \\ k_{1m1}\\ \vdots \\ k_{x1}\\ k_{x2}\\ \vdots \\ k_{xm_{x}}\\ \vdots \\ k_{m1}\\ k_{m2}\\ \vdots \\ k_{mm_{m}} \end{array} & {\left[\begin{array}{ccccc} O_{k}^{b11} & \cdots & O_{k}^{b1y} & \cdots & O_{k}^{b1m}\\ \vdots & & \vdots & & \vdots \\ O_{k}^{bx1} & \cdots & O_{k}^{bxy} & \cdots & O_{k}^{bxm}\\ \vdots & & \vdots & & \vdots \\ O_{k}^{bm1} & \cdots & O_{k}^{bmy} & \cdots & O_{k}^{\rho mm} \end{array}\right]}_{z\times z|m<z,\sum_{y=1}^{m}m_{y}=z} \end{array}$$

$O_{k}^{b11}$, as a normalized instance, determines how to normalize activities for the fundamental concept, as shown in Equations (A9) and (A10):

(A9) $$v_{x}^{11}=\sum_{y=1}^{m_{1}}o_{xy}^{11},x=1,2,\ldots ,m_{1}$$

(A10) $$O_{k}^{b11}=[\begin{array}{ccccc} O_{11}^{11}/v_{1}^{11} & \cdots & O_{1y}^{11}/v_{1}^{11} & \cdots & O_{1m1}^{11}/v_{1}^{11}\\ \vdots & & \vdots & & \vdots \\ O_{x1}^{11}/v_{i}^{11} & \cdots & O_{xy}^{11}/v_{x}^{11} & \cdots & O_{im1}^{11}/v_{x}^{11}\\ \vdots & & \vdots & & \vdots \\ O_{m_{1}1}^{11}/v_{m_{1}}^{11} & \cdots & O_{m_{1}y}^{11}/v_{m_{1}}^{11} & \cdots & O_{m_{1}m_{1}}^{11}/v_{m_{1}}^{11} \end{array}]=[\begin{array}{ccccc} O_{11}^{\alpha 11} & \cdots & O_{1y}^{\alpha 11} & \cdots & O_{1m_{1}}^{\alpha 11}\\ \vdots & & \vdots & & \vdots \\ O_{x1}^{\alpha 11} & \cdots & O_{xy}^{\alpha 11} & \cdots & O_{xm_{1}}^{\alpha 11}\\ \vdots & & \vdots & & \vdots \\ O_{m_{1}1}^{\alpha 11} & \cdots & O_{m_{1}y}^{\alpha 11} & \cdots & O_{m_{1}m_{1}}^{\alpha 11} \end{array}]$$

Secondly, the normalized matrix $O_{k}^{b}$ of the full-influence relationship is altered to obtain the super-matrix $W={\left(O_{k}^{b}\right)}^{\prime }$ which is unweighted, as shown in Equation (A11):

(A11) $$W={\left(O_{k}^{b}\right)}^{\prime }=\begin{array}{ccc} & & \begin{array}{ccccc} V_{1} & & V_{x} & & V_{m}\\ k_{11}\ldots k_{1m_{1}} & \cdots & k_{x1}\ldots k_{xm_{x}} & \cdots & k_{m1}\ldots k_{mm_{m}} \end{array}\\ \begin{array}{c} V_{1}\\ \vdots \\ V_{y}\\ \vdots \\ V_{m} \end{array} & \begin{array}{c} k_{11}\\ k_{12}\\ \vdots \\ k_{1m_{1}}\\ \vdots \\ k_{y1}\\ k_{y2}\\ \vdots \\ k_{ym_{y}}\\ \vdots \\ k_{\alpha 1}\\ k_{\alpha 2}\\ \vdots \\ k_{mm_{m}} \end{array} & {\left[\begin{array}{ccccc} W^{11} & \cdots & W^{x1} & \cdots & W^{m1}\\ \vdots & & \vdots & & \vdots \\ W^{1y} & \cdots & W^{xy} & \cdots & W^{my}\\ \vdots & & \vdots & & \vdots \\ W^{1m} & \cdots & W^{x\alpha } & \cdots & W^{mm} \end{array}\right]}_{z\times z|m<z,\sum_{y=1}^{m}m_{y}=z} \end{array}$$

### Appendix B.2. Stage 2: Creating a Super-Matrix Weight

The super-matrix weight $W^{*}$ is able to be separated into two stages: the initial act normalizes the matrix $O_{V}$ of the full influence relationship (Equation (A5)), and transposes it to obtain the normalized matrix $O_{V}^{b}$ of the full influence relationship, as shown in Equations (A12) and (A13). Then, the normalized matrix $O_{V}^{b}$ of the full influence relationship is multiplied by the super-matrix unweighted W, which can determine weighted super-matrix $W^{*}$, as shown in Equation (A14):

(A12) $$v_{x}=\sum_{y=1}^{m}O_{v}^{xy},x=1,2,\ldots ,m\;\mathrm{and}\;o_{V}^{bxy}=f_{D}^{xy}/v_{x},y=1,2,\ldots ,m$$

(A13) $$O_{v}^{b}={\left[\begin{array}{ccccc} O_{v}^{11}/v_{1} & \cdots & O_{v}^{1y}/v_{1} & \cdots & O_{v}^{1m}/v_{1}\\ \vdots & & \vdots & & \vdots \\ O_{v}^{x1}/v_{x} & \cdots & O_{v}^{xy}/v_{x} & \cdots & O_{v}^{im}/v_{x}\\ \vdots & & \vdots & & \vdots \\ O_{v}^{m1}/v_{m} & \cdots & O_{v}^{my}/v_{m} & \cdots & O_{v}^{mm}/v_{m} \end{array}\right]}_{m\times m}={\left[\begin{array}{ccccc} O_{v}^{b11} & \cdots & O_{v}^{b1y} & \cdots & O_{v}^{b1m}\\ \vdots & & \vdots & & \vdots \\ O_{v}^{bx1} & \cdots & O_{v}^{bxy} & \cdots & O_{v}^{bxm}\\ \vdots & & \vdots & & \vdots \\ O_{v}^{bm1} & \cdots & O_{v}^{bmy} & \cdots & O_{v}^{bmm} \end{array}\right]}_{m\times m}$$

(A14) $$W^{b}=O_{V}^{b}\times W=\left[\begin{array}{ccccc} o_{V}^{b11}\times W^{11} & \cdots & o_{V}^{bx1}\times W^{x1} & \cdots & o_{V}^{bm1}\times W^{m1}\\ \vdots & & \vdots & & \vdots \\ o_{V}^{b1y}\times W^{1y} & \cdots & o_{V}^{bxy}\times W^{xy} & \cdots & o_{V}^{bmy}\times W^{mj}\\ \vdots & & \vdots & & \vdots \\ o_{V}^{b1m}\times W^{1m} & \cdots & o_{V}^{bxm}\times W^{xm} & \cdots & o_{V}^{bmm}\times W^{mm} \end{array}\right]$$

### Appendix B.3. Stage 3: Assembling the Supermatrix Weight

This paper applies the Makov chain procedure of ANP to assemble the super-matrix weight $W^{*}$ via itself several times till the supermatrix was converted into a steady supermatrix with an adequately large power Θ. Therefore, the influence ratio levels of the factors are attained by $\underset{\Theta \to \infty }{\lim}{\left(W^{v}\right)}^{\Theta }$. Lastly, we acquire a series of influence weights for factors $\left(w_{1},\ldots ,w_{j},\ldots ,w_{n}\right)$ and dimensions $\left(w_{1}^{b},\ldots ,w_{j}^{b},\ldots ,w_{m}^{b}\right)$.

## Appendix C. Modified VIKOR Technique

We define the modified VIKOR technique, as follows.

Stage 1: indicates the levels $x_{j}^{*}$ and $x_{j}^{-}$ in quality factor assessment. The level $x_{j}^{*}$ denotes the positive-ideal point, which is the best score for factor *j*; and $x_{j}^{-}$ denotes the negative-ideal point, which is the worst score for factor *j*. $x_{kj}$ represents the performance score of *k* alternative for *j* factor. The enhancement of the modified VIKOR technique is initiated by the next procedure of the $L_{p}$_metric:

(A15) $$L_{k}^{p}={\left\{\sum_{j=1}^{n}{\left[w_{j}(\left|x_{j}^{*}-x_{kj}\right|)/(\left|x_{j}^{*}-x_{j}^{-}\right|)\right]}^{p}\right\}}^{1/p}$$

As 1≤p≤∞;k=1,2,…,m and it influences weight, $w_{j}$ is obtained by DANP. This study applied the novel concepts of Equations (A16) and (A17) to acquire the outcomes for the development gaps per dimension/factor according to feedback and interdependence issues:

$$x_{j}^{*}=\underset{k}{\max}\;x_{kj},j=1,2,\ldots ,n$$

We set the aspired values, vector

(A16) $$x^{*}=(x_{1}^{*},x_{2}^{*},\cdots ,x_{n}^{*})$$

$$x_{j}^{-}=\underset{k}{\min}\;x_{kj},j=1,2,\ldots ,n$$

We set the worst values, vector

(A17) $$x^{-}=(x_{1}^{-},x_{2}^{-},\cdots ,x_{n}^{-})$$

In the paper, we used questionnaires in which the scored responses range from 0 to 10: totally dissatisfied (0) to extremely satisfied (10). The fundamental concept of this technique is to apply performance scores according to experts; thus, the worst value is set at zero and the aspired level is set at 10. Hence, we set $x_{j}^{*}=10$, *j* = 1,2, …, *n* as the aspired level and $x_{j}^{-}=0$, *j* = 1,2, …, *n* as the worst value, which differs from the traditional method. In this method, we use $x_{j}^{*}$ as aspired value and $x_{j}^{-}$ as the worst value, in order to prevent selecting the best from among inferior alternatives.

Stage 2: indicates the minimum mean of cluster utility $F_{k}$ and maximal regret $Q_{k}$:

(A18) $$L_{k}^{p=1}=F_{k}=\sum_{j=1}^{n}w_{j}r_{kj}=\sum_{j=1}^{n}w_{j}\left(\left|x_{j}^{*}-x_{kj}\right|\right)/\left(\left|x_{j}^{*}-x_{j}^{-}\right|\right)$$

(A19) $$L_{k}^{p=\infty }=S_{k}=\underset{j}{\max}\left\{r_{kj}|j=1,2,\ldots ,n\right\}$$

where $r_{kj}=\left(\left|x_{j}^{*}-x_{kj}\right|\right)/\left(\left|x_{j}^{*}-x_{j}^{-}\right|\right)$ indicates the ratio of the gap; $F_{k}$ indicates the average gap ratios through aspirated value $x_{j}^{*}$ to performance value $x_{kj}$ in factor *j* of alternative *k*, and this study focused on how to minimalize gap $r_{kj}$ for all factors j=1,2,…,n. Formerly, $w_{j}$ indicated the relative influence weight of factor *j*; $w_{j}$ can be determined through DANP according DEMATEL. $Q_{k}$ indicates the maximum gap for all factors.

Stage 3: indicates comprehensive factor $R_{k}$ and its various consequences. Equation (A20) computes the values. From Equation (A20), we can detect how to enhance the adoption of an e-health cloud computing system, in order to decrease the gaps in reaching the desired values according to the influence relationship map:

(A20) $$R_{k}=v\left(F_{k}-F^{*}\right)/\left(F^{-}-F^{*}\right)+\left(1-v\right)\left(S_{k}-S^{*}\right)/\left(S^{-}-S^{*}\right)$$

This study applied the levels obtained by $S^{*}=\underset{k}{\min}S_{k}$ or $F^{*}=0$, $F^{-}=\underset{k}{\max}F_{k}$ or $F^{-}=1$; $S^{*}=\underset{k}{\min}S_{k}$ or $S^{*}=0$), $S^{-}=\underset{k}{\max}S_{k}$ or $S^{-}=1$. Therefore, the gap for $S^{*}=0$, $F^{-}=1$, $S^{*}=0$, and $S^{-}=1$; thus, we can re-write Equation (A20) as $R_{k}=vF_{k}+(1-v)S_{k}$. Weight v=1 only indicates how to reduce the average gap, while weight v=0 only decides how to choose the largest gap for enhancement. Generally, v=0.5 can be used to according to this condition.

According to the above concepts and applying the influential network relationship map, we can simply attain how to enhance these gaps *r_kj_* (*k* = 1, 2, …, *m*; *j* = 1, 2, …, *n*), that is, the enhancement sequence to attain the aspired value.
